# Estrogen receptor α gene polymorphism and vascular complications in girls with type 1 diabetes mellitus

**DOI:** 10.1007/s11010-017-3103-0

**Published:** 2017-06-20

**Authors:** Bartosz Słomiński, Jolanta Myśliwska, Monika Ryba-Stanisławowska, Maria Skrzypkowska, Małgorzata Myśliwiec

**Affiliations:** 1 Department of Immunology, Medical University of Gdańsk, ul. Dębinki 1, 80-211 Gdańsk, Poland; 2 Chair & Clinics of Paediatrics, Diabetology and Endocrinology, Medical University of Gdańsk, Dębinki 7, 80-211 Gdańsk, Poland

**Keywords:** Diabetes-related vascular complications, Estrogen receptor α gene polymorphism, Inflammation, CD16, Serum lipid profile, Angiogenic factors

## Abstract

The effect of estrogens is mediated by activation of estrogen receptors (ERs). Because *ER*-*α* gene polymorphisms may exert different effects in childhood, we analyzed the associations between the IVS1 −397T>C (PvuII) polymorphism and systemic inflammatory state, proangiogenic factors, frequency of monocyte subsets, lipid profile, blood pressure, and vascular complications in girls with type 1 diabetes mellitus (DM1). We examined 180 young girls with DM1 and 120 healthy age-matched controls. The analysis concerned PvuII polymorphism of the *ER*-*α* gene as well as the levels of serum inflammatory markers (CRP, IL-6, TNF-α), proangiogenic factors (VEGF, angiogenin), 17β-estradiol, values of monocyte subsets (CD14^++^CD16^−^ and CD14^+^CD16^+^), lipid profile, and blood pressure. In our study, girls with CC genotype had lower level of inflammatory and angiogenic factors and lower frequencies of CD14^+^CD16^+^ monocytes in comparison to CT or TT carriers. Simultaneously, the CC carriers had a greater population of CD14^++^CD16^−^ monocytes, increased blood pressure, and serum levels of: estrogen, total cholesterol, triglycerides, and low-density lipoprotein cholesterol than girls bearing CT or TT genotype. Our study suggests a pleiotropic effect of PvuII polymorphic CC variant on diabetic vasculopathies. Although the CC genotype carriers demonstrate less inflammatory and angiogenic activity, they seem to display less favorable cardiometabolic features. Based on our study, we cannot distinguish PvuII *ER*-*α* genotype that could be useful in identification of DM1 girls that are more prone to develop of late vascular complications, before the occurrence of first clinical symptoms.

## Introduction

Type 1 diabetes mellitus (DM1) is caused by autoimmune selective destruction of pancreatic β-cells. As a multifactorial disease, it is caused by a complex combination of genetic and environmental factors triggering autoimmunity [1]. Diverse genetic polymorphisms may influence individual’s susceptibility to DM1 or severity of illness once symptoms occur [2]. Identification of genetic factors associated with diabetes may lead to primary prediction of disease development and prevention of its vascular complications, such as hypertension, retinopathy, and nephropathy. The importance of such approach is associated with retinopathy and nephropathy being major causes of blindness and end-stage renal failure in diabetic individuals. Furthermore, vascular complications remain the major cause of morbidity and mortality in this population [3].

Diabetes-related vascular complications share etiological characteristics. Factors recognized as responsible for the pathogenesis of diabetic vasculopathy are: hyperglycemia, hyperlipidemia, growth factors, hormones, and inflammation [4]. Low grade chronic systemic inflammation underlies DM1 and plays a crucial role in the development of late microvascular complications [5]. Furthermore, the onset of microangiopathy is associated with infiltration by inflammatory cells, as well as elevated levels of CRP and proinflammatory cytokines [6]. Infiltration by neutrophils and monocytes, firm adhesion of these leukocytes to vascular endothelial cells are one of the earliest events present for many years before overt retinopathy or nephropathy [7, 8]. Other risk factors for the onset and progression of diabetic complications are hyperlipidemia and overall altered lipid metabolism, as various studies have proven significant relevance of lipid abnormalities to vascular complications in DM1 [9, 10].

Accumulating experimental and clinical data confirmed the influence of gender on autoimmunity, with women being much more susceptible to autoimmune disorders [11]. Moreover, females with DM1 have roughly 40% greater risk of all-cause mortality and are twice as likely to suffer from fatal or nonfatal vascular events when compared with DM1 males [12], indicating the importance of sex hormones in the development of diabetes and its succeeding complications. Thus, factors involved in response to sex hormones stimulation are logical candidates to assess their possible connections with susceptibility to DM1 and the overall course of the disease.

Estrogens play an important role in the development and function of male and female reproductive systems. They also exert effects on the immune system [13], skeletal system, lipid metabolism, as well as the maintenance and protection of cardiovascular and nervous systems [14]. Therefore, it is evident that estrogens impact certain physiological and metabolic disorders including: insulin resistance, dyslipidemia, and hypertension [15]. The effect of estrogens is mainly mediated by binding to and activation of estrogen receptors (ER-α and ER-β). A number of observational clinical studies have analyzed the associations between polymorphic variants of *ER*-*α* gene and estrogen-related clinical effects [16]. The most studied variations are PvuII (IVS1 −397T>C, rs2234693) and XbaI single nucleotide polymorphism (SNP) localized in the first intron of *ER*-*α* gene. Functional mechanisms attributed to the polymorphisms of the *ER*-*α* gene include alternation of transcription factors binding sites followed by modified gene expression [17] or a change in mRNA processing, leading to the production of multiple variants or isoforms of the protein [18].

Some studies suggested a link between PvuII polymorphic variants of the *ER*-*α* gene and inflammation as well as vascular complications in DM1 patients. In our previous study [19], we have shown that diabetic girls carrying CC genotype were characterized by greater population of CD4^+^Foxp3^+^ Tregs and simultaneous lower TNF serum level in comparison to girls carrying CT or TT genotype. Moreover, elevated levels of analyzed proinflammatory factors (Th17 cells, cytokines) in DM1 girls carrying TT genotype promoted enhanced inflammatory response, which led to development and progression of diabetic microvascular complications [20]. In another study conducted on DM1 boys, researchers have discovered that individuals carrying CC genotype had lower blood pressure, IL-6, and CRP serum levels. Similar results were obtained in boys with DM1 and microvascular complications [21].

Some genetic investigations were conducted to establish the effect of PvuII *ER*-*α* gene polymorphism on systemic inflammation. Some studies indicated proinflammatory profile in individuals with TT variant of this polymorphism [22], whereas other showed lack of such connection [23] or even inverse relationship between presence of this particular genotype and chosen inflammation-related factors [18]. Despite their differences, these studies have provided further evidence of the potential role of PvuII *ER*-*α* gene polymorphism in inflammation. Our previous studies indicated that CC variant of PvuII *ER*-*α* gene polymorphism may be connected with attenuated chronic inflammatory response during DM1 [19–21]. However, a relationship between this polymorphism and vascular complications in different sex groups still remains unclear.

Taking all these into account, we aimed to examine whether the PvuII *ER*-*α* gene polymorphism is associated with the major contributory factors for vascular complications such as systemic inflammatory state, lipid profile, proangiogenic factors, blood pressure, and vascular complications in girls with DM1.

## Materials and methods

### Participants

This case–control study evaluated 180 young regularly menstruating girls with diagnosed type 1 diabetes who were recruited from the Chair and Clinics of Pediatrics, Diabetology and Endocrinology, Medical University of Gdańsk. Mean age of patients was 15.5 ± 3.5 years. The diagnosis of type 1 diabetes was based on the American Diabetes Association criteria [24]. Patients with coexisting autoimmune, chronic, and acute, inflammatory diseases were excluded from the study. All patients were treated with humanized insulin at doses of 0.87 ± 0.2 U/kg. At the time of sampling, lipid levels (total cholesterol—TC, triglycerides—TG, high-density lipoprotein cholesterol—HDL, low-density lipoprotein cholesterol—LDL) along with biochemical measurement of renal function, C-reactive protein (CRP) and glycated hemoglobin (HbA1c) was monitored.

The control group consisted of age and BMI matched 120 healthy young menstruating girls recruited during control visits in an outpatient clinic. No signs of autoimmune, chronic, inflammatory, or neoplastic disease at the time of sampling and no evidence of DM1 in their families were disclosed as confirmed by medical records, laboratory examination and laboratory tests.

The urinary albumin excretion (UAE) was expressed as the average of three 24 h collections. Cases were classified as microalbuminuria when in at least two out of three urine samples, UAE ratio was >30–300 mg/24 h. Diabetic nephropathy was defined as persistent microalbuminuria in two out of three consecutive urine samples without clinical or laboratory evidence of other kidney or urinary tract disease.

All of the girls with diabetes-related vascular complications (hypertension, nephropathy, and retinopathy) were newly diagnosed and previously untreated.

The blood from all patients was collected in the follicular phase (between days two and four) of menstrual cycle. Additionally, the level of plasma 17β-estradiol was determined in all girls using commercial enzyme-linked immunosorbent assay kits (R&D Systems, Minneapolis, Minn., USA) according to the manufacturer’s protocol.

The study was conducted according to the principles of the Declaration of Helsinki. All individuals provided written informed consent to participate in a study. The protocol was approved by the Ethics Committee of the Medical University of Gdańsk.

### Sample collection

Serum samples were collected from all girls and processed by centrifugation at 500×*g* for 15 min and stored at −70 °C until analysis.

Genomic DNA from all the subjects was isolated from EDTA-stabilized blood using the EXTRACTME DNA BLOOD (Blirt, Poland). DNA was stored at −20 °C until the time of use.

### Medical examinations

Systolic and diastolic blood pressures (SBP and DBP, resp.) were measured using automatic 24 h ambulatory blood pressure monitoring (ABPM) by the Holter method. All the average values of the blood pressure were expressed in the centile charts. Arterial hypertension was diagnosed when the blood pressure value reached at least 95th percentile for the corresponding age, gender, and height on at least three separate occasions [25].

Ophthalmologic investigation was performed in all girls with DM1. Diabetic retinopathy was determined by visual acuity, intraocular pressure measurement, anterior segment estimation by slit lamp (TOPCON SL-82, Japan), and fluorescein angiography (digital camera-Topcon IMAGEnet2000, Japan). The eye fundus examination was performed with the +90D lens (Ocular Instruments Inc., Bellevue, Wash, USA). Analysis of the eye fundus pictures was based on The International Diabetic Retinopathy Division [26].

### SNP genotyping

Characterization of IVS1 −397T>C of the ER-α genotypes (rs2234693) was analyzed as previously described [21].

DNA samples were first sequenced to establish three ER-α gene polymorphic variants, as a quality control. Next, DNA samples of the CC, CT, and TT individuals were routinely added to the examined ones to ensure genotype accuracy.

### Measurements of inflammation markers and angiogenic factors

Plasma cytokines were measured at the time of inclusion in the study. Levels of IL-6, TNF-α, VEGF, and angiogenin were determined using commercial enzyme-linked immunosorbent assay kits (R&D Systems, Minneapolis, Minn., USA) according to the manufacturer’s protocol.

### Flow cytometric staining and analysis

By means of flow cytometry, the values of CD14^++^CD16^−^ and CD14^+^CD16^+^ monocytes in the peripheral blood of all subjects were evaluated. 50 μL aliquots of fresh venous blood were stained with anti-CD14 (IgG2b mouse PerCP, clone MφP9; BD Bioscences, USA) and anti-CD16 (IgG2 mouse APC-Cy7, clone 3G8, BD Bioscences) human antibodies. For each set, appropriate isotype control was done. The samples were incubated for 30 min in the dark at room temperature, lysed, and fixed with Immuno-prep reagents (Immunotech, USA) on Q-prep Immunology Workstation (Coulter, USA).

Expression of cell surface markers was assessed using flow cytometry (LSRII, BD Bioscences) and data were analyzed by FACSDiva 6.0 Software (BD Bioscences). Monocytes were gated according to their forward and side scatter characteristics. Typically, 10,000 events were acquired in this region. The monocytes subsets were identified based on the expression of the CD14 and CD16 molecules from dot plots representing CD14 versus CD16 staining.

### Statistical analysis

The results were analyzed using Statistica, ver. 12 (StatSoft, Inc., USA). Conformation of the allele frequencies to the Hardy–Weinberg equilibrium proportions was tested by the χ^2^ test. Normally distributed variables were analyzed with the one-way ANOVA test. Nominal variables were analyzed by the χ^2^ Pearson test. For comparison of the nonparametric variables, the Kruskal–Wallis ANOVA test was applied. The level of significance was set at *p* ≤ 0.05.

## Results

### The IVS1 −397T>C estrogen receptor α genotype distribution

The frequencies of IVS1 −397T>C genotypes in DM1 group were as follows: CC—23.9%, CT—45%, TT—31.1%. These frequencies conformed to the Hardy–Weinberg equilibrium (*p* = 0.20). The genotype distributions in the group of healthy girls were: CC—22.5%, CT—52.5%, TT—25% and they also conformed to the Hardy–Weinberg principle (*p* = 0.58). Comparison of frequencies of PvuII genotypes between the DM1 patients and healthy group revealed lack of significant differences (*p* = 0.40; χ^2^ Pearson test).

### The IVS1 −397T>C ER-α gene polymorphism and clinical characteristics of patients

The clinical characteristics of diabetic patients differing in the IVS1 −397T>C estrogen receptor α polymorphism are presented in Table 1. Girls with different IVS1 −397T>C estrogen receptor α genotypes did not reveal significant differences in: age, duration of disease, BMI, HbA1c, urinary albumin excretion, and serum creatinine level. However, we have noticed that in this group the values of systolic and diastolic blood pressure did differ between variants of *ER*-*α* gene (*p* = 0.003 and *p* = 0.02, resp.). Systolic blood pressure was the highest in patients with CC genotype and the lowest in TT carriers. Similarly, CC variants of *ER*-*α* gene had significantly higher diastolic blood pressure when compared to CT and TT bearing patients.

**Table 1:** Clinical characteristics of girls with DM1 differing in the IVS1 −397T>C estrogen receptor α polymorphism

Clinical parameter	IVS1 −397T>C			*p*
	CC	CT	TT	
*N* (%)	43 (23.9)	81 (45.0)	56 (31.1)	–
Age (years)	15.6 ± 3.8	15.8 ± 3.5	15.2 ± 4.4	0.75
Duration of diabetes (years)	6.4 ± 2.5	5.9 ± 2.6	6.2 ± 3.3	0.58
BMI (kg/m^2^)	20.2 ± 2.6	19.4 ± 2.8	19.6 ± 3.3	0.37
HbA1c (%)	8.7 ± 1.9	8.6 ± 1.8	8.9 ± 1.7	0.61
Albumin excretion rate (mg/24 h)	15.8 ± 15.2	17.7 ± 14.0	20.2 ± 17.7	0.37
Serum creatinine level (mg/dL)	0.75 ± 0.10	0.74 ± 0.13	0.72 ± 0.12	0.47
17β-Estradiol (pg/mL)	66.3 ± 17.7	52.3 ± 18.3	46.6 ± 14.9	**<0.001**
Systolic blood pressure (mmHg)	116 ± 9	110 ± 10	111 ± 10	**0.003**
Diastolic blood pressure (mmHg)	74 ± 7	71 ± 8	70 ± 6	**0.02**

Differences were calculated by the ANOVA test. Data are presented as arithmetic mean ± standard deviation (SD)

Bold *p* value indicates that *p* is lower than 0.05

*p*—the comparison between three analyzed genotypes: CC, CT, and TT

*N* number of patients

As to estrogen concentration, the level of 17β-estradiol was measured in serum of DM1 girls, with samples being collected between 2nd and 4th days of their menstrual cycle. We have noticed that DM1 girls possessing CC genotype had higher estradiol levels (*p* < 0.001) when compared to CT and TT carriers.

### Serum levels of inflammation markers and angiogenic factors according to genetic variant of the IVS1 −397T>C ER-α gene polymorphism

To extend our analysis, we measured the serum levels of inflammation markers (CRP, IL-6, TNF-α) and angiogenic factors (VEGF, angiogenin) in DM1 girls with different genotypes (Table 2). We found that CC carriers had lower levels of CRP, IL-6, and TNF-α compared to their CT and TT counterparts (*p* = 0.003, *p* = 0.004 and *p* < 0.001, resp.). Moreover, the CC variants of *ER*-*α* gene had lower VEGF and angiogenin levels when compared with CT and TT patients (*p* = 0.005 and *p* < 0.001, resp.).

**Table 2:** Serum levels of inflammation markers and angiogenic factors in girls with DM1 differing in the IVS1 −397T>C estrogen receptor α polymorphism

Parameter	IVS1 −397T>C			*p*
	CC	CT	TT	
CRP (mg/mL)	1.56 ± 1.19	1.77 ± 1.32	2.66 ± 2.46	**0.003**
IL-6 level (pg/mL)	0.82 ± 1.24	1.44 ± 1.31	1.57 ± 0.80	**0.004**
TNF-α level (pg/mL)	0.68 ± 0.61	1.18 ± 0.92	1.30 ± 0.84	**<0.001**
VEGF (pg/mL)	102 ± 157	119 ± 136	187 ± 132	**0.005**
Angiogenin (ng/mL)	430 ± 120	532 ± 161	566 ± 164	**<0.001**

Differences were calculated by the ANOVA test. Data are presented as arithmetic mean ± standard deviation (SD)

Bold *p* value indicates that *p* is lower than 0.05

*p*—the comparison between three analyzed genotypes: CC, CT, and TT

### Monocyte subsets among the IVS1 −397T>C ER-α genotypes

By means of flow cytometry, we identified two monocyte subsets in DM1 girls: the CD14^++^CD16^−^ and CD14^+^CD16^+^ cells (Table 3). Analyzing CD14^++^CD16^−^ monocytes, among peripheral blood mononuclear cells, girls bearing the CC genotype had the highest frequency of these cells (Table 3). Moreover, the CC genotype variant group was found to have the lowest frequency of CD14^+^CD16^+^ (Table 3).

**Table 3:** The frequency of CD14^++^CD16^−^ and CD14^+^CD16^+^ cells in girls with DM1 differing in the IVS1 −397T>C estrogen receptor α polymorphism

	IVS1 −397T>C			p*
	CC	CT	TT	
CD14^++^CD16^−^ (%)	93.2 ± 3.4	92.8 ± 3.2	88.1 ± 2.9	**<0.001**
CD14^+^CD16^+^ (%)	6.9 ± 1.6	7.2 ± 1.8	11.8 ± 2.0	**<0.001**

Differences were calculated by the ANOVA test. Data are presented as arithmetic mean ± standard deviation (SD)

Bold *p* value indicates that *p* is lower than 0.05

* The comparison between three analyzed genotypes: CC, CT, and TT

### The IVS1 −397T>C ER-α gene polymorphism and lipid profile in girls with DM1

The next step of our work was to analyze the association between lipid levels (total cholesterol—TC, triglycerides—TG, high-density lipoprotein cholesterol—HDL, low-density lipoprotein cholesterol—LDL) and the genetic variant of estrogen receptor α gene polymorphism (Table 4). We found that girls with DM1 bearing CC genotype had higher levels of TC, TG, and LDL (*p* = 0.001; *p* < 0.001 and *p* = 0.004, resp.) but not HDL (*p* = 0.26) when compared to their CT and TT counterparts.

**Table 4:** Lipid profile in girls with DM1 differing in the IVS1 −397T>C estrogen receptor α polymorphism

Parameter	IVS1 −397T>C			*p*
	CC	CT	TT	
Cholesterol	5.09 ± 1.08	4.50 ± 0.87	4.49 ± 0.71	**0.001**
Triglycerides	1.44 ± 1.06	1.01 ± 0.43	0.97 ± 0.41	**<0.001**
HDL	1.49 ± 0.25	1.55 ± 0.31	1.59 ± 0.31	0.26
LDL	2.91 ± 0.82	2.48 ± 0.77	2.46 ± 0.65	**0.004**

All the values are in mmol/L

Differences were calculated by the ANOVA test. Data are presented as arithmetic mean ± standard deviation (SD)

Bold *p* value indicates that *p* is lower than 0.05

*p*—the comparison between three analyzed genotypes: CC, CT, and TT

### The IVS1 −397T>C genotype distribution in DM1 girls with macro- and microvascular complications

The group of girls with type 1 diabetes was analyzed with regard to hypertension as existing macrovascular complication. The results are shown in Table 5. There were no differences between genotypes of *ER*-*α* gene with respect to hypertension occurrence (*p* = 0.21).

**Table 5:** The IVS1 −397T>C genotype distribution in DM1 girls with macrovascular complication—hypertension

IVS1 −397T>C genotypes	Girls with DM1 (*N* = 180)		Girls with DM1 without hypertension (*N* = 157)		Girls with DM1 and hypertension (*N* = 23)		χ^2^ Pearson
	*N*	%	*N*	%	*N*	%	
CC	43	23.9	38	24.2	5	21.7	3.12 *p* = 0.21*
CT	81	45.0	67	42.7	14	60.9	
TT	56	31.1	52	33.1	4	17.4	

* Significance between DM1 girls with and without hypertension

Among 180 diabetic girls, 31 patients had nephropathy and 22 had retinopathy (Tables 6, 7). However, we did not observe alterations in the frequencies of IVS1 −397T>C *ER*-*α* genotypes due to diabetic microvascular complications development. Alleles distribution in patients with nephro- and retinopathy was similar in comparison to complications-free group (*p* = 0.48 and *p* = 0.31, resp.).

**Table 6:** Distribution of IVS1 −397T>C genotypes in DM1 girls with microvascular complications—nephropathy

IVS1 −397T>C genotypes	Girls with DM1 (*N* = 180)		Girls with DM1 without nephropathy (*N* = 149)		Girls with DM1 and nephropathy (*N* = 31)		χ^2^ Pearson
	*N*	%	*N*	%	*N*	%	
CC	43	23.9	37	24.8	6	19.3	1.47 *p* = 0.48*
CT	81	45.0	64	42.9	17	54.8	
TT	56	31.1	48	32.3	8	25.9	

* Significance between DM1 girls with and without nephropathy

**Table 7:** Distribution of IVS1 −397T>C genotypes in DM1 girls with microvascular complications—retinopathy

IVS1 −397T>C genotypes	Girls with DM1 (*N* = 180)		Girls with DM1 without retinopathy (*N* = 158)		Girls with DM1 and retinopathy (*N* = 22)		χ^2^ Pearson
	*N*	%	*N*	%	*N*	%	
CC	43	23.9	40	25.3	3	13.6	2.33 *p* = 0.31*
CT	81	45.0	68	43.0	13	59.1	
TT	56	31.1	50	31.7	6	27.3	

* Significance between DM1 girls with and without retinopathy

## Discussion

The increased incidence of autoimmune disorders among female patients and a greater risk of fatal and nonfatal vascular events in DM1 women gave rise to an interest in the regulation of the immune/inflammatory response by sex hormones. In the present study, we analyzed the association of the PvuII *ER*-*α* gene polymorphism with systemic inflammatory state, lipid profile, proangiogenic factors, blood pressure, and vascular complications in girls with type 1 diabetes mellitus.

Similarly to previous reports regarding PvuII *ER*-*α* gene polymorphism in a smaller group of girls [19, 20] and boys [21] with DM1, researchers have found that CC genotype is associated with weakened inflammatory response. CC genotype bearing girls produced more 17β-estradiol and had lower CRP, IL-6, and TNF-α serum level than their counterparts with CT and TT genotype. Herrington et al. [27] have proven that the CC variant of the PvuII *ER*-*α* gene polymorphism binds estrogens much more efficiently than TT, which is consistent with studies suggesting that lower estrogen concentration is associated with an increased production of proinflammatory cytokines [28, 29].

In this study, we have noticed that girls with DM1 bearing CC genotype had higher systolic and diastolic blood pressure in comparison to CT and TT genotype bearing counterparts. Interestingly, we have already observed an inverse relationship between PvuII *ER*-*α* gene polymorphism and blood pressure in DM1 boys, where CC genotype was connected with the lowest values of SBP and DBP [21]. This observation is significant as higher values of SBP and DBP are associated with faster progression of late diabetic complications [30]. PvuII *ER*-*α* gene polymorphism may influence blood pressure regulation since estrogens are known to stimulate vasodilation, mainly by inducing production of nitric oxide [31] in an ER-α dependent manner [32]. Moreover, the N-terminus of ER-α plays a major role in activation of ER-α relative genes and alterations in this region have been linked to hypertension [33, 34].

Another important observation from the current study is the fact, that girls with DM1 carrying CC genotype had lower VEGF and angiogenin serum level when compared to their CT and TT genotype bearing counterparts. VEGF is a critical component in tissue growth and organ repair processes mediated by angiogenesis and vasculogenesis. As a factor included in blood vessel formation, it also plays a pivotal role in the development of microvascular complications. In some disorders, elevated VEGF level acts as a pathological angiogenic stimulus (i.e., ocular neovascularization), whereas in others, low protein activity leads to other undesirable conditions (i.e., cardiomyopathy, peripheral neuropathy) [35]. Angiogenin is one of the most potent stimulators of vascular growth [36]. High angiogenin level induces excessive angiogenesis, which in turn may be responsible for diabetic nephropathy progression by causing enlargement of the glomerular filtration surface [37]. Surprisingly, in young patients with DM1, regardless of the extent of microvascular complications, increased angiogenin levels have been denoted [38, 39].

By means of flow cytometry, we have identified two monocyte subsets in DM1 girls: the “classical”—CD14^++^CD16^−^ and “nonclassical”—CD14^+^CD16^+^ cells. We have found that girls bearing the CC genotype had higher frequencies of classical monocytes and lower frequencies of nonclassical cells when compared with CT and TT counterparts. During infection, classical monocytes, comprising ~85% of the whole monocyte population undergo rapid recruitment from the blood stream into surrounding tissues, where they become activated. Nonclassical monocytes are localized within capillaries, small veins, and arteries where they crawl over the endothelium patrolling the vasculature for tissue damage or infection. The CD16^+^ monocytes undergo expansion in various infectious and inflammatory diseases as well as are involved in pathogenesis of autoimmune disorders [40]. In addition, these cells are chief producers of TNF-α, releasing this cytokine preferentially on the layer of endothelial cells and not spilling it over to the blood stream [41]. Based on these features, CD16^+^ cells were termed proinflammatory monocytes [42]. Estrogens modulate the expression of CD16 by a mechanisms that can utilize ERs, including the interaction of ER-α with the CD16 promoter. This causes change in a profile of released cytokines upon receptor activation [43]. Based on our results and literature [5, 44, 45] it is highly probable that CD16^+^ monocytes play an important role in promoting inflammatory response in DM1.

Another observation from the current study is the fact, that girls with DM1 bearing CC genotype had higher levels of TC, TG, and LDL but not HDL when compared to their CT and TT counterparts. Various studies have shown a positive correlation between elevated serum lipids (TC, TG, LDL) and macrovascular complications [46]. On the other hand, studies investigating association between elevated serum lipids and microvascular complications have shown varying results [47]. Despite their differences, these studies offer evidence for an important role of various lipid abnormalities in the development and progression of vascular diabetic complications. Estrogens influence hepatic expression of genes involved in lipoprotein metabolism, which results in increased serum HDL and TG concentrations but decreased serum LDL level [32]. Even though previous findings on the association of PvuII *ER*-*α* gene polymorphism with lipid profile are controversial, there are clear proofs that PvuII polymorphism can influence estrogen-dependent lipid variables [48].

In the present study, the PvuII ER-α genotype distribution within patients with vascular diabetic complications showed lack of statistically significant differences when compared to uncomplicated patients. We can speculate that CC variant of this polymorphism is associated with weaker inflammatory response and angiogenic activity leading to slower progression of diabetic complications. On the other hand, CC variant promotes cardiometabolic risk factors responsible for more rapid progression of vascular dysfunctions in DM1. Taking all of these facts into account, this is possibly the reason why we cannot observe differences in PvuII ER-α genotypes distribution in DM1 girls with and without vascular complications. The list of risk factors examined in our study is not exhaustive and other components may influence the association between PvuII polymorphism and cardiovascular diabetic complications.

In conclusion, at this stage of our study, it is hard to clearly determine whether PvuII ER-α genotype polymorphism may be a useful tool for identification of late vascular complications, as its CC variant seems to reduce inflammation which protects against development of complications. On the other hand, the same CC variant seems to impair lipid profile in DM1 patients making them more prone to develop cardiovascular complications.
